# Arbutin Alleviates LPS Induced Sepsis Pneumonia in Mice

**DOI:** 10.1155/2022/5863952

**Published:** 2022-04-16

**Authors:** Xiang-Xiang Bian, Xuan Zhao, Chun-Hua Ma, Chuan-Pu Shen

**Affiliations:** ^1^School of Pharmacy, Anhui Medical University, Hefei 230032, China; ^2^The Experimental Center of the First Clinical Medical College, Nanjing University of Chinese Medicine, Nanjing 210000, China

## Abstract

The aim of this study was to investigate the effects of arbutin (AR) on lipopolysaccharide (LPS)-induced sepsis pneumonia. LPS-induced mice and A549 cells were used to establish septic pneumonia model. AR significantly decreased lung wet-to-dry weight (W/D) ratio, lung myeloperoxidase (MPO) activity and ameliorated lung histopathological changes. In addition, AR increased super oxide dismutase (SOD) activity, decreased malondialdehyde (MDA) content and levels of cytokines including tumor necrosis factor-*α* (TNF-*α*), interleukin-1*β* (IL-*β*) and interleukin-6 (IL-6) in bronchoalveolar lavage fluid (BALF) in mice. Furthermore, the results demonstrated that AR inhibited the JAK2/STAT3/NF-*κ*B pathway in LPS-induced A549 cells which was further confirmed by siRNA JAK2 experiment. The experimental results indicated that the protective mechanism of AR on sepsis pneumonia might be attributed partly to the inhibition of cytokine production and JAK2/STAT3/NF-*κ*B pathway.

## 1. Introduction

Acute lung injury (ALI) refers to the injury of alveolar epithelial cells and pulmonary capillary endothelial cells caused by non-cardiogenic factors. The pathological characteristics include impairment of alveolar capillary barrier, infiltration of inflammatory cells in the lung, diffuse alveolar and interstitial edema. Its main pathophysiological characteristics are decreased lung compliance, lung volume, imbalance of ventilation/blood flow ratio, and clinical manifestations are respiratory distress and refractory hypoxemia. If the ALI cannot be controlled, it can further develop to its severe stage, acute respiratory distress syndrome (ARDS) [1]. Although some progress has been made in the diagnosis and treatment of ALI/ARDS in recent years, the disease still has a relatively high mortality rate [2]. The mortality rate of ALI patients is 38.5%, while the mortality rate of ARDS patients is as high as 41.1% [3]. Therefore, more effective drugs are urgently needed in clinical treatment to control symptoms and reduce disease mortality. A variety of etiologies can induce the onset of Ali, such as sepsis, pneumonia, inhalation of gastric contents, severe trauma, acute pancreatitis and blood transfusion [4]. Among these pathogenic factors, sepsis caused by Gram-negative bacteria is the most common pathological state of ALI/ARDS [5]. Lipopolysaccharide (LPS) from Gram-negative bacteria is believed to play a key role in the inflammatory response of ALI [6]. Based on this, we used LPS to induce inflammatory reaction in vitro and used LPS to induce ALI model in mice. Arbutin, a hydroquinone glucoside hydroquinone, is a natural active substance extracted from different plants [7–9]. It has certain specific physiological functions and exists widely in animal, plant and microbial cells [10]. There are few reports on the effect of sepsis pneumonia. The purpose of this experiment is to study the effect of AR on sepsis pneumonia.

## 2. Materials and Methods

### 2.1. Reagent

LPS and AR were purchased from Sigma Company, Cytokine detection ELISA kit was purchased from American R&D company. The biochemical index detection kit is a product of Nanjing Jiancheng Biological Co., Ltd. BCA protein detection kit is a product of Shanghai Beyotime Biological Co., Ltd. All antibodies were purchased from Cell Signaling Technology.

### 2.2. Cell Source and Culture

A549 cells purchased from American Cell Center. A549 cells were inoculated in DMEM medium containing 10% fetal bovine serum (containing 100 IU/mL penicillin and 100 IU/mL).

### 2.3. Animals and Modeling

Healthy adult male BABL/c mice, weighing 20–22 g, were purchased from Shanghai Slake Experimental Co., Ltd. and fed in a clean-grade animal house, with free access to food and water. After 1 week of adaptive feeding, the mice were randomly divided into control group, ALI group, AR and dexamethasone (Dex) treatment group, 10 mice in each group. After intraperitoneal injection of pentobarbital sodium (50 mg/kg), the mice were bluntly separated to expose the trachea. The ALI group and the AR treatment group were injected with LPS (8 mg/kg, 50 *μ*L) to replicate the ALI animal model. Mice were injected with an equal volume of saline. The AR and Dex treatment group was injected intraperitoneally with AR (20, 40 mg/kg) and Dex (2 mg/kg) for 30 minutes before LPS injection, and the remaining groups were injected with saline. After 6 hours of modeling, 4 mice from each group were killed and lung tissues were taken for HE staining and gene expression detection. Eight additional mice from each group were taken for bronchoalveolar lavage. All animal experiments were conducted in accordance with the Ethics Committee of Health Medical Research of Anhui Medical University (NO. 20180321).

### 2.4. A549 Cells Transfection

A549 cells were cultured in DMEM medium (containing 10% fetal bovine serum, 100 mg/L streptomycin and 0.1 U/L ampicillin) at 37°C and 5% CO_2_. 24 hours before transfection, 7 × 10^5^ A549 cells were seeded on a six-well plate overnight. When the cell density was 60%, pHBV1.1 (4 *μ*g) was transfected into A549 cells by liposome 2000 reagent. In the siRNA experiment, the JAK2 siRNA sequence is 5′-GATCAATGGCTACACAGGA-3′, and the negative control siRNA sequence is 5′-TCCCGAACGTGTCACGT-3′. siRNA was transfected into the primary cancer cells and other cells with Lipofectamine 2000 reagent. 48 hours after transfection, total RNA and protein samples were taken for detection.

### 2.5. Establishment of A549 Cells Inflammation Model Induced by LPS

A549 cells with stable passage and good growth status were taken, the cell suspension concentration was adjusted, and seeded in 6-well plates and 96-well plates respectively, and the cells were cultured for 12 h under the conditions of 37°C and 5% CO_2_. After taking out, discard the supernatant, wash twice with PBS, add medium and LPS with a final concentration of 2 *μ*g/L to the 6-well plate and 96-well plate, and put it back into the incubator at 37°C, 5% CO_2_. The cell culture was continued for 12 h under the conditions, and the inflammation model was successfully established. Randomly divided into 6 groups, namely: normal group, model group (LPS group), AR group (10, 20, 40 *μ*M), siRNA-JAK2 + AR (40 *μ*M), each group was set up 3 complex wells. The LPS treatment group was pre-incubated with LPS with a final concentration of 2 *μ*g/mL for 4 h, and the AU treatment group was given AR with a final concentration of 10, 20, and 40 *μ*M, respectively.

### 2.6. Bronchoalveolar Lavage in Mice

After the mice were anesthetized and fixed, the exposed trachea was bluntly separated, inserted into the trachea with an indwelling needle and ligated, 0.8 mL of cold saline was injected into the lung, slowly aspirated 3 times, and finally recovered and placed on ice, repeated Second, the total fluid collected is bronchoalveolar lavage fluid (BALF).

### 2.7. Detection of Wet to dry Weight Ratio (W/D) of Lung Tissue

Six hours after modeling, the right lung of the mouse was taken out, placed on tin foil, and weighed with a microbalance to record the data. The weight of lung tissue minus the weight of tin foil was the wet weight of the lung tissue of the mouse. The lung tissue of the mice in the group was wrapped with tin foil, and then the wrapped lung tissue was placed in a constant temperature oven and continuously baked at 85°C for 48 h. After 48 h, it was taken out, weighed, and the data was recorded. The weight minus the weight of tin foil is the dry weight of lung tissue. The result of dividing the wet weight of the lung tissue by the dry weight is the wet to dry weight ratio of the lung tissue of the mouse.

### 2.8. MPO Detection

Mice lung tissue was obtained, using 1 : 9 saline homogenate, centrifuged to obtain supernatant, using commercial reagents to detect MPO content, while using BCA kit to detect protein content, thereby obtaining lung tissue MPO content.

### 2.9. Detection of SOD Activity and MDA Content in BALF and Cell Supernatant

The SOD activity and MDA level of BALF and cell supernatant were tested using commercial kits, and the operation method was carried out according to the kit instructions.

### 2.10. Detection of TNF-*α*, IL-6 and IL-1*β* in BALF and Cell Supernatant

Cytokines in BALF and cell supernatant were also detected by ELISA kit. The specific method and steps of the experiment were tested according to the experimental guidance given by R&D.

### 2.11. HE Staining

The upper lobe of the left lung of the mouse was fixed in 4% paraformaldehyde. After paraffin embedding, 4 *μ*m serial sections were taken and HE staining was performed by conventional methods. According to the method reported earlier by the research group, the pathological damage score of lung tissue in mice.

### 2.12. Western Blotting

The lung tissue obtained from the experimental mice in each group was first rinsed gently with pre-chilled 1 × PBS buffer twice, then the lung tissue was placed in 2 ml EP, 500 *μ*L of protein lysis solution was added, and homogenized with a tissue homogenizer After the plasma lung tissue is marked, it was then centrifuged at 4°C and 12000 rpm for 10 minutes using a low-temperature high-speed centrifuge. After centrifugation, the supernatant was sucked out and placed in a 1.5 mL EP tube and marked on ice. Then the total protein content was detected by BCA kit. After cleaning the glass plate and comb, mix with glue, mix with 1 × electrophoresis solution, and adjust the voltage to 60 V after adding the sample. We will prepare the wet transfer solution and transfer the film before electrophoresis, the condition is 300 mA, 90 minutes; prepare the blocking solution in advance, After the membrane transfer is completed, place the membrane in the blocking solution for 1 hour, dilute the antibody concentration accordingly according to the instructions of the antibody, and incubate the primary antibody overnight on the shaker; the next day, wash the membrane 3 times with 1 × TBST prepared in advance. Approximately 15 minutes each time, incubate the secondary antibody after washing, incubate for 2-3 hours on a shaker and wash the membrane again 3 times with 1 × TBST for approximately 15 minutes each time; add ECL developer according to the size of the membrane and incubate at room temperature 30 s, adjust the best exposure time according to the signal strength during development.

### 2.13. Statistical Processing

Measurement data was expressed as mean ± standard deviation (*x* ± *s*), and statistical analysis was performed using SPSS 17.0. ANOVA was used to compare the mean between multiple groups of normal distribution data, and the SNK-*q* test was used to compare each group. *P* < 0.05 means the difference is statistically significant.

## 3. Results

### 3.1. The Effects of AR on MPO in Lung Tissue in ALI Mice

**Figure 1:**
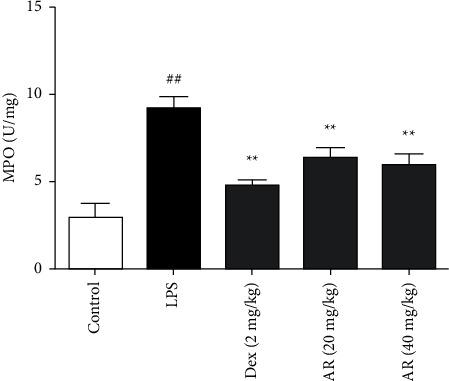
The effects of AR on MPO in lung tissue in ALI mice (*n* = 10). MPO is detected by commercial kit, which is strictly in accordance with the kit instructions. Values are expressed as means ± SD. Compared with control: ^##^*P* < 0.01; Compared with LPS: ^*∗∗*^*P* < 0.01.

MPO is an important indicator of ALI. Therefore, Therefore, this experiment first tests this index. Compared with normal group, MPO content of lung tissue in ALI group was significantly increased. Compared with ALI group, AR and Dex significantly reduced MPO content in lung (Figure 1).

### 3.2. The Effects of AR on W/D in ALI Mice

**Figure 2:**
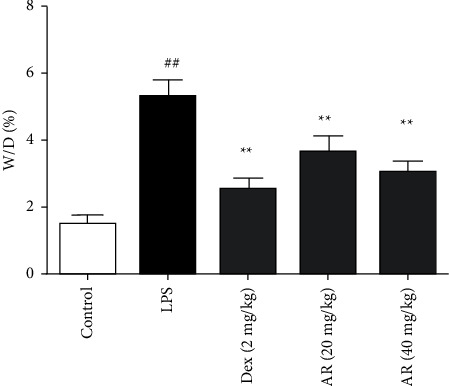
The effects of AR on W/D in ALI mice (*n* = 10). The result of dividing the wet weight of the lung tissue by the dry weight is the wet to dry weight ratio of the lung tissue of the mouse. Values are expressed as means ± SD. Compared with control: ^##^*P* < 0.01; Compared with LPS: ^*∗∗*^*P* < 0.01.

W/D is one of the most basic and important methods in lung examination, which is of great significance to the diagnosis of lung diseases. Compared with control group, W/D in ALI group was increased. Compared with ALI group, AR and Dex significantly reduced W/D (Figure 2).

### 3.3. The Effects of AR on SOD Activity and MDA Content in BALF and Cell Supernatant

**Figure 3:**
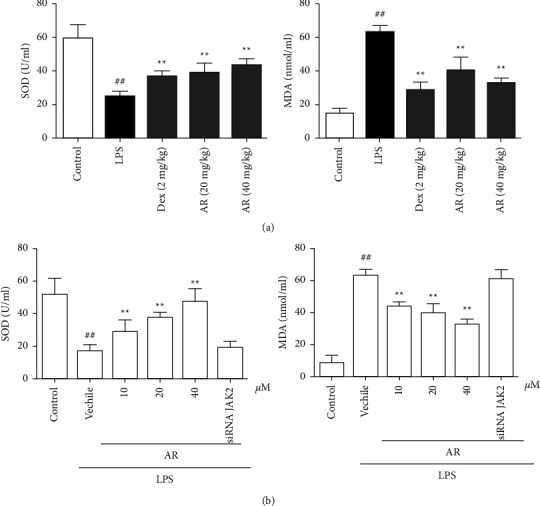
The effects of AR on SOD activity and MDA content in BALF (a) and cell supernatant (b) (*n* = 10). SOD and MDA are detected by commercial kit, and the method is according to the kit instructions. Values are expressed as means ± SD. Compared with control: ^##^*P* < 0.01; Compared with LPS: ^*∗∗*^*P* < 0.01.

The oxidative stress in lung is unbalanced when ALI occurs. Therefore, the determination of oxidative stress index is an important index to evaluate lung injury. For BALF, compared with control group, SOD activity was significantly decreased and MDA content was significantly decreased in LPS group. AR and Dex significantly increased SOD and decreased MDA (Figure 3(a)). For cell supernatant, compared with control group, SOD activity was significantly decreased and MDA content was significantly decreased in LPS group. AR and Dex significantly increased SOD and decreased MDA, siRNA JAK2 canceled the above changes (Figure 3(b)).

### 3.4. The Effects of AR on TNF-*α*, IL-6 and IL-1*β* in Mouse BALF and Cell Supernatant

**Figure 4:**
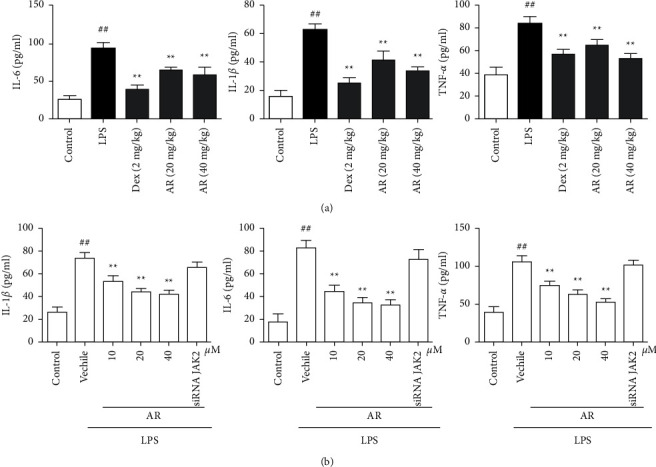
The effects of AR on TNF-*α*, IL-6 and IL-1*β* in BALF (a) and cell supernatant (b) (*n* = 10). TNF-*α*, IL-6 and IL-1*β* were detected by commercial kits, and the operation method was carried out according to the kit instructions. Values are expressed as means ± SD. Compared with control: ^##^*P* < 0.01; Compared with LPS: ^*∗∗*^*P* < 0.01.

Cytokines play a very important role in the process of immune response, and become one of the hot spots of ALI research at present because of their participation in the process of immune response and inflammatory response. For BALF, compared with control group, TNF-*α*, IL-6 and IL-1*β* levels was significantly increased in LPS group. AR and Dex significantly decreased TNF-*α*, IL-6 and IL-1*β* levels (Figure 4(a)). For cell supernatant, compared with control group, TNF-*α*, IL-6 and IL-1*β* levels were significantly increased in LPS group. AR and Dex significantly decreased TNF-*α*, IL-6 and IL-1*β* levels, siRNA JAK2 canceled the above changes (Figure 4(b)).

### 3.5. HE Staining

**Figure 5:**
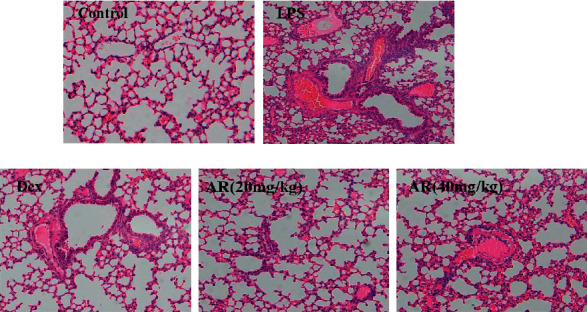
HE staining (x200) (*n* = 6). Values are expressed as means ± SD. Compared with control: ^##^*P* < 0.01; Compared with LPS: ^*∗∗*^*P* < 0.01. White arrows indicate inflammatory infiltration, the red arrow indicates lung atrophy, blue arrow indicates pulmonary edema.

After 6 hours of LPS, a large amount of alveolar collapse, inflammatory cell infiltration, and pulmonary edema were seen in the tissue, and the pathological injury score of the lung tissue increased significantly. AR and Dex treatment significantly reduced the lung tissue pathological damage in ALI mice, and the lung tissue pathological damage score was decreased (Figure 5).

### 3.6. The Effects of AR on JAK2/STAT3 Pathway and NF-*κ*B in Mice and A549 Cells

**Figure 6:**
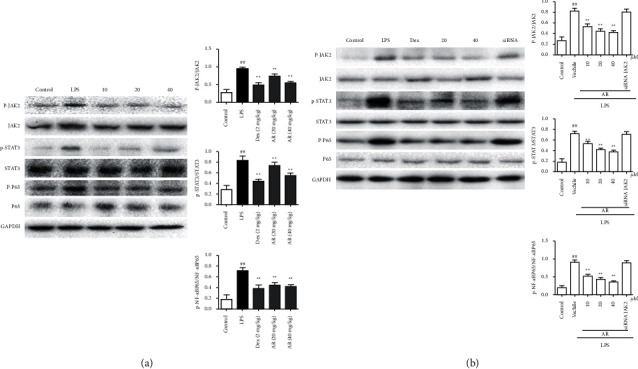
The effects of AR on JAK2/STAT3/NF-*κ*B pathway in mice (a) and A549 cells (b) (*n* = 10). JAK2/STAT3/NF-*κ*B pathway was detected by western blot. Values are expressed as means ± SD. Compared with control: ^##^*P* < 0.01; Compared with LPS: ^*∗∗*^*P* < 0.01.

For mice, compared with control group, p-JAK2, p-STAT3 and p-NF-*κ*BP65 levels was significantly increased in LPS group. AR and Dex significantly decreased p-JAK2, p-STAT3 and p-NF-*κ*BP65 (Figure 6(a)). For A549 cells, compared with control group, p-JAK2, p-STAT3 and p-NF-*κ*BP65 levels was significantly increased in LPS group. AR and Dex significantly decreased p-JAK2, p-STAT3 and p-NF-*κ*BP65, siRNA JAK2 canceled the above changes (Figure 6(b)).

## 4. Discussion

Acute lung injury and its severe form of acute respiratory distress syndrome (ARDS) remain the leading cause of death in the intensive care unit (ICU). The typical characteristics of acute lung injury are pulmonary edema, inflammation, intrapulmonary hemorrhage, and severe gas exchange disorders [11, 12]. In many cases, such as sepsis, pancreatitis, multiple trauma, pneumonia, lung transplantation or inhalation of harmful gases may cause acute lung injury [13]. Although low tidal volume ventilation, early neuromuscular blockade, and prone position can reduce ARDS mortality, the overall mortality of ARDS in ICU and hospitals is still maintained [14]. To date, there are no drugs that can effectively reduce the mortality of adults with ARDS. This study found that AR treatment significantly reduced LPS-induced lung weight and lung/body weight ratio, improved alveolar edema, cell necrosis and inflammatory cell infiltration, reduced cytokine expression, improved lung pathological changes, and suppressed lung JAK2/STAT3/NF-*κ*B pathway.

The ideal animal model should be able to replicate the pathogenesis and outcome of human diseases, including physiological and pathological features. LPS is the main component of the cell wall of Gram-negative bacteria. After entering animals and humans, LPS can cause damage to the microvascular endothelial cells of epithelial cells in lung tissue, including the accumulation of leukocytes in lung tissue, pulmonary edema, and cascade-amplified inflammation [15]. LPS is not only a common cause of direct lung damage (such as pneumonia), but also a common cause of indirect lung damage (such as sepsis). It can also cause other chronic diseases. The methods that can be used to induce acute lung injury using LPS include intranasal instillation, intratracheal instillation, intraperitoneal injection and intravenous injection. Intranasal instillation and intratracheal infusion of LPS can cause acute lung injury within 24 hours of administration, while intravenous or intraperitoneal injection of LPS does not cause tissue-specific or similar degrees of lung injury. Therefore, we used the method of direct administration into mice trachea to induce lung damage. Our research found LPS could successfully induce a model of acute lung injury in mice.

The pathophysiology of acute lung injury/acute respiratory distress syndrome includes inflammation with diffuse cell injury, increased capillary permeability plus, interstitial edema and influx of circulating inflammatory cells [16]. Pro-inflammatory cytokines such as interleukin-1*β* and tumor necrosis factor-*α* are significantly elevated in patients with acute lung injury/acute respiratory distress syndrome. This study found that AR treatment significantly reduced LPO-induced MPO, W/D, improved alveolar edema, cell necrosis and inflammatory cell infiltration, and reduced cytokine expression in mice.

At present, it is considered that an important factor in the development of sepsis pneumonia disease is the imbalance of the immune system, which includes the destruction of the balance of T cell subsets and the accumulation of local pro-inflammatory cytokines to increase inflammation. The most critical signaling pathways involved in the apoptosis, function and cytokine function of T cell proliferation and differentiation are JAK2/STAT3/NF-*κ*B pathway [17–19]. JAK/STAT signal pathway is an important cytokine signal transduction pathway, which plays a vital role in human physiology and pathology, immune regulatory response. Current studies have shown that a large number of external stimulus signals may lead to activation of NF-*κ*B signaling pathways, including lipopolysaccharides [20, 21]. The primary function of I*κ*B protein is to prevent NF-*κ*B protein from entering the nucleus and binding to DNA, so that NF-*κ*B protein remains in the cytoplasm. Therefore, the study of I*κ*B protein is particularly important in exploring the mechanism of NF-*κ*B signaling pathway [22]. Western blot analysis indicated that the protective mechanisms of AR might be related to the inhibition of JAK2/STAT3/NF-*κ*B pathway, siROCK1 counteracted the above changes. In summary, AR has a certain reversal effect on LPS-induced lung oxidative damage and inflammation, which is related to AR's inhibition of JAK2/STAT3/NF-*κ*B pathway.

## Data Availability

There is no underlying data need to be found.

## Abbreviations

AR: Arbutin
LPS: Lipopolysaccharide
W/D: Wet-to-dry weight
MPO: Myeloperoxidase
SOD: Super oxide dismutase
MDA: Malondialdehyde
TNF-*α*: Tumor necrosis factor-*α*
IL-1*β*: Interleukin-1*β*
IL-6: Interleukin-6
BALF: Bronchoalveolar lavage fluid
ALI: Acute lung injury.
